# The 2023 *Nucleic Acids Research* Database Issue and the online molecular biology database collection

**DOI:** 10.1093/nar/gkac1186

**Published:** 2023-01-06

**Authors:** Daniel J Rigden, Xosé M Fernández

**Affiliations:** Institute of Systems, Molecular and Integrative Biology, University of Liverpool, Crown Street, Liverpool L69 7ZB, UK; IQVIA Ltd., The Point, 37 North Wharf Road, London W2 1AF, UK

## Abstract

The 2023 *Nucleic Acids Research* Database Issue contains 178 papers ranging across biology and related fields. There are 90 papers reporting on new databases and 82 updates from resources previously published in the Issue. Six more papers are updates from databases most recently published elsewhere. Major nucleic acid databases reporting updates include Genbank, ENA, ChIPBase, JASPAR, mirDIP and the Issue's first Breakthrough Article, NACDDB for Circular Dichroism data. Updates from BMRB and RCSB cover experimental protein structural data while AlphaFold 2 computational structure predictions feature widely. STRING and REBASE are stand-out updates in the signalling and enzymes section. Immunology-related databases include CEDAR, the second Breakthrough Article, for cancer epitopes and receptors alongside returning IPD-IMGT/HLA and the new PGG.MHC. Genomics-related resources include Ensembl, GWAS Central and UCSC Genome Browser. Major returning databases for drugs and their targets include Open Targets, DrugCentral, CTD and Pubchem. The EMPIAR image archive appears in the Issue for the first time. The entire database Issue is freely available online on the *Nucleic Acids Research* website (https://academic.oup.com/nar). The NAR online Molecular Biology Database Collection has been updated, revisiting 463 entries, adding 92 new resources and eliminating 96 discontinued URLs so bringing the current total to 1764 databases. It is available at http://www.oxfordjournals.org/nar/database/c/.

## NEW AND UPDATED DATABASES

In its 30th incarnation, the *Nucleic Acids Research* Database Issue once again ranges across biology with a total of 178 papers. Table 1 lists the 90 new databases included, a recent record number, and there are 82 update papers from resources previously covered by NAR. Finally, six databases most recently published elsewhere contribute updates (Table 2). As usual, updates from the major database providers at the European Bioinformatics Institute (EBI), the U.S. National Center for Biotechnology Information (NCBI), and the National Genomics Data Center (NGDC) in China (1–3) are placed first. The usual categorisation then follows: (i) nucleic acid sequence and structure, transcriptional regulation; (ii) protein sequence and structure; (iii) metabolic and signalling pathways, enzymes and networks; (iv) genomics of viruses, bacteria, protozoa and fungi; (v) genomics of human and model organisms plus comparative genomics; (vi) human genomic variation, diseases and drugs; (vii) plants and (viii) other topics, such as proteomics databases. Many papers are not easily placed in a single category so readers are well advised to browse the full list.

**Table 1.** Descriptions of new databases in the 2023 NAR database Issue

Database name	URL	Short description
ABC Portal	http://abc.sklehabc.com	Single cell transcriptomics of blood cells
AgeAnno	https://relab.xidian.edu.cn/AgeAnno/#/	Single cell annotation of aging in human
Amylograph	http://AmyloGraph.com/	Amyloid-amyloid interactions
Animal-SNPAtlas	http://gong_lab.hzau.edu.cn/Animal_SNPAtlas/	High-quality SNPs in 20 animal species
Antibody Registry	https://antibodyregistry.org	Antibodies, their antigens and catalogue numbers
Aquila	https://aquila.cheunglab.org	Spatial omics data
ASCancer Atlase	https://ngdc.cncb.ac.cn/ascancer/home	Alternative splicing in cancer
BIC	http://bic.jhlab.tw/	Bacteria in Cancer
Brain Catalog	https://ngdc.cncb.ac.cn/braincatalog	Genetics of brain disorders and related phenotypes
BV-BRC	https://www.bv-brc.org/	Bacterial and Viral Bioinformatics Resource Center
CEDAR	https://cedar.iedb.org/	Cancer Epitope Database and Analysis Resource
Cell Taxonomy	https://ngdc.cncb.ac.cn/celltaxonomy	Cell types and markers across species, tissues and conditions
Cell Tracer	http://bio-bigdata.hrbmu.edu.cn/CellTracer	Multi-omics of cellular development trajectories
ChemFOnt	https://www.chemfont.ca	Chemical Functional Ontology
ChemPert	https://chempert.uni.lu/	Transcriptomics responses to pertubagens in non-cancerous cells
CMDB	https://db.cngb.org/cmdb/	Whole-genome sequencing data of 141,000 Chinese individuals
ChromLoops	https://3dgenomics.hzau.edu.cn/chromloops	Protein-mediated chromatin loops
CohesinDB	https://cohesindb.iqb.u-tokyo.ac.jp	Multi-omics data on cohesin functions
COMBATdb	https://db.combat.ox.ac.uk	COVID-19 Multi-omics Blood Atlas
CottonMD	http://yanglab.hzau.edu.cn/CottonMD/	Multi-omics data on cotton
CovInter	https://idrblab.org/covinter	Interactions between coronavirus RNAs and host proteins
CRAMdb	http://www.ehbio.com/CRAMdb	Microbiome metagenomes across animals
CRdb	http://cr.liclab.net/crdb/	Human chromatin regulators
CREAMMIST	https://creammist.mtms.dev	Cancer drug dose-reponse across cell lines
CRISPRbase	http://crisprbase.maolab.org	CRISPR Base Editing
DirectRMDB	http://www.rnamd.org/directRMDB/	RNA modifications from direct RNA sequencing
DRESIS	https://idrblab.org/dresis/	Drug resistance
DrugMAP	https://idrblab.org/drugmap/	Molecular Atlas and Pharma-information of drugs
DupScan	https://dupscan.sysumeg.com/	Vertebrate genome duplications
EDomics	http://edomics.qnlm.ac	Comparative multi-omics for animal Evo-Devo
EmAtlas	http://bioinfor.imu.edu.cn/ematlas	Spatiotemporal multi-omics of mammalian embryogenesis
FAVOR	https://favor.genohub.org	Functional Annotation of Variants - Online Resource
Fungal Names	https://nmdc.cn/fungalnames/	Fungal taxonomy
G4Atlas	https://www.g4atlas.org/	Experimentally determined RNA G-quadruplexes in transcriptomes
GAIA	https://gaia.cobius.usherbrooke.ca	Predicted G-quadruplexes in genomes and transcriptomes
GenomicKB	https://gkb.dcmb.med.umich.edu/	Knowledge Graph for the Human Genome
GotEnzymes	https://metabolicatlas.org/gotenzymes	Predicted enzyme kinetic parameters
GPSAdb	https://www.gpsadb.com/	Genetic Perturbation Similarity Analysis
HGD	https://ngdc.cncb.ac.cn/hgd	Homolog Gene Database – expression, traits, variants across species
HiChIPdb	http://health.tsinghua.edu.cn/hichipdb/	HiChIP regulatory interactions
HProteome-BSite	https://galaxy.seoklab.org/hproteome-bsite/database	Predicted binding sites and ligands in the human 3D proteome
HTCA	https://www.htcatlas.org	Single cell transcriptomes and analytical tools
HUSCH	http://husch.comp-genomics.org	Human Universal Single Cell Hub
IAnimal	https://ianimal.pro/	Multi-omics data for 21 animals
ipaQTL	http://bioinfo.szbl.ac.cn/iaQTL/	Intronic polyadenylation quantitative trait loci
IEAtlas	http://bio-bigdata.hrbmu.edu.cn/IEAtlas	HLA-presented immune epitopes from non-coding regions etc
ImmCluster	http://bio-bigdata.hrbmu.edu.cn/ImmCluster	Immunology cell types in normal and cancer tissues
IntroVerse	https://rytenlab.com/browser/app/introverse	Introns and splicing errors/noise
isomiRdb	https://anathema.cs.uni-saarland.de/isomirdb/	microRNA isoform expression
Lineage Landscape	http://data.iscr.ac.cn/lineage/#/home	Omics of lineage determination in animals
M6AREG	https://idrblab.org/m6areg/	m6A vs disease and drug response
MeDBA	https://medba.ddtmlab.org	Metalloenzyme families, structures, ligands
MediaDive	https://mediadive.dsmz.de	Culture media
MHC Motif Atlas	http://mhcmotifatlas.org/	MHC Binding Specificities and Ligands
microbioTA	http://bio-annotation.cn/microbiota	Cancer microbiomes
MiMeDB	https://mimedb.org	Human-microbe interactions and their metabolomes
NACDDB	https://genesilico.pl/nacddb/	Nucleic Acid Circular Dichroism Database
NEMO Archive	https://nemoarchive.org	Neuroscience Multi-Omic Archive
NLRscape	https://nlrscape.biochim.ro/	Plant Nod-like receptors
OrganoidDB	http://www.inbirg.com/organoid_db/	Organoid transcriptomics
PAT	http://bioinfo.qd.sdu.edu.cn/PAT/	Prokaryotic Antimicrobial Toxin database
PDCM Finder	www.cancermodels.org	Patient-Derived Cancer Models
PGG.MHC	https://pog.fudan.edu.cn/pggmhc	Population genetics of HLA genes
PGG.SV	https://www.biosino.org/pggsv	Human genome structural variants
PertOrg	http://www.inbirg.com/pertorg/	Changes induced in model organisms by in vivo genetic perturbation
plantEXP	https://biotec.njau.edu.cn/plantExp	Plant gene (co-)expression and alternative splicing
ProPan	https://ngdc.cncb.ac.cn/propan	Prokaryotic pan-genomes
ProtCAD	http://dunbrack2.fccc.edu/protcad	Protein Common Assembly Database
qPTM	http://qptm.omicsbio.info	Quantitative Post-Translational Modifications
QUADRAtlas	https://rg4db.cibio.unitn.it	RNA G-Quadruplex and RG4-binding proteins
RABC	http://www.onethird-lab.com/RABC/	Multi-omics of Rheumatoid Arthritis
RBPimage	http://rnabiology.ircm.qc.ca/RBPImage/	Microscopy reporting subcellular distribution of human RNA binding proteins
Ribocentre	https://www.ribocentre.org	Ribozymes
RiboXYZ	https://ribosome.xyz	Ribosome structures
Ribo_uORF	http://rnainformatics.org.cn/RiboUORF	uORFs from ribosome profiling data
RLBase	https://gccri.bishop-lab.uthscsa.edu/shiny/rlbase/	R-loops in the human genome
RM2Target	http://rm2target.canceromics.org/	Targets of RNA modification proteins
SPASCER	https://ccsm.uth.edu/SPASCER	Spatial transcriptomics annotation at single-cell resolution
SPEED	http://speedatlas.net	Single-cell pan-species atlas
SUMMER	http://njmu-edu.cn:3838/SUMMER/	Biomarkers, GWAS and cancer survival
tatDB	https://grigoriev-lab.camden.rutgers.edu/tatdb	Experimentally supported targets of tRNA-derived fragments
TEDD	https://TEDD.obg.cuhk.edu.hk/	Temporal Expression of Development Database
TFSyntax	https://tfsyntax.zhaopage.com	Transcription Factor binding syntax
Thing Metabolome Repository family	http://metabolites.in/things	LC–MS metabolomics data
TIMEDB	https://timedb.deepomics.org	RNAseq data and the tumor immune micro-environment
TmAlphaFold	https://tmalphafold.ttk.hu/	AlphaFold TM protein predictions, membrane-embedded and assessed
tModBase	https://www.tmodbase.com/	tRNA modifications: enzymes, dynamics, diseases
TOXRIC	http://toxric.bioinforai.tech/	Toxicological data and benchmarks
TWAS Atlas	https://ngdc.cncb.ac.cn/twas/	Transcriptome-wide association studies
UFCG	https://ufcg.steineggerlab.com	Fungal core genes

**Table 2.** Updated descriptions of databases most recently published elsewhere

Database name	URL	Short description
Chemical Probes Portal	https://www.chemicalprobes.org/	Chemical probe assessment and selection
CIViC	https://civicdb.org/	Clinical interpretation of variants in cancer
EMPIAR	https://empiar.org/	The Electron Microscopy Public Image Archive
EpiFactors	http://epifactors.autosome.org	Human epigenetic factors and complexes
FerrDb	http://www.zhounan.org/ferrdb/	Ferroptosis regulators
SulfAtlas	https://sulfatlas.sb-roscoff.fr/	Sulfatase families

The ‘Nucleic acid databases’ section contains the first of the Issue's Breakthrough Articles, reporting on the Nucleic Acid Circular Dichroism Database (NACCDDB; (4)). CD data can give insights into the folding, stability, dynamics and interactions of nucleic acids. NACDDB archives and disseminates the experimental spectra for the first time alongside the metadata describing the experiment and any associated structure models. At this early stage, the database is keen to receive new data and feedback directly from the community. A trio of new nucleic acid quadruplex-related databases also feature. G4Atlas (5) focuses on experimentally determined RNA G-quadruplexes (rG4s) across transcriptomes, determined by a variety of experimental methods, and accompanied by their classification into canonical and other types. QUADRAtlas (6) similarly focuses on rG4s, covering both experimental and predicted structures and including information on rG4-binding proteins, while GAIA (7) surveys predicted quadruplexes in both genomes and transcriptomes across all three kingdoms. RNA modifications are also addressed by three new databases. tModBase (8) focuses on modifications of tRNA, their dynamics and biomedical implications, and the enzymes involved. RM2Target (9), a development of the earlier m6A2Target database (10), covers writes, erasers and readers of nine RNA modifications as well as diverse annotations and biomedical implications. The third of the trio, DirectRMDB (11), collects data from direct RNA sequencing that captures quantitative RNA modifications, annotating the results in an isoform-specific manner. Long ncRNA data are covered in update papers from three popular resources. lncRNASNP (12) reports hugely expanded content and a variety of new analyses and annotations, many focusing on diseases especially cancer, while LncTarD (13) for experimentally-supported lncRNA-target interactions more than doubles in size and introduces new features, again including some focused on cancer. The third, LncBook (14), a curated database of human lncRNAs, reports improved multi-omics annotations including, for the first time, any experimentally-supported small proteins that they encode. Two further databases focus on uORFs: uORFDB (15) returns and expands on its original literature focus to now cover, sequences and sequence variants across 13 eukaryotes, while the new Ribo_uORF (16) provides rich annotations of uORFs identified by ribosome profiling across six animal species. Elsewhere, popular returning databases include AnimalTFDB which doubles its content to cover 183 animals (17); mirDIP, the aggregative database of microRNA–target interactions (18) and UTRdb, the database of richly annotated 5′ and 3′ untranslated regions of mRNA (19).

The reverberations of the AlphaFold 2 (AF2) earthquake (20,21) continue to be felt across the database community. In the protein section, a new database TmAlphaFold (22) addresses the fact that AF2 has no explicit knowledge of the position of the lipid bilayer in which many proteins are embedded. By predicting the membrane embedding of models in the AlphaFold Protein Structure Database (AFDB; (23)) the new resource allows valuable extra validation of transmembrane helical protein models. Another new database, HPproteome-BSite (24), annotates predicted binding sites and candidate ligands across AFDB models of the human proteome. The returning AlloMAPS database (25) now encompasses AFDB entries and other new-generation structure predictions, offering improved insights into the impact of mutations on allostery and helping design of allosteric drugs. The RCSB Protein Data Bank (26) reports that structure predictions, including AFDB, are now available via its website, alongside its core experimentally determined structures which now number almost 200 000. The update from GPCRdb (27) reports state-specific AF2 models alongside other new features such as lists of ligands for each receptor, both endogenous and surrogate. Even MobiDB (28), focusing on intrinsically disordered proteins, benefits from two AF2-related predictions unforeseen by the original methods developers: predictions of disordered regions and potential interaction motifs contained within them. Other classes of proteins with special structural properties are covered by the new Amylograph (29) which curates information on amyloid-amyloid interactions and the returning PhaSepDB (30) for proteins that can participate in phase separation, now doubled in size and with much more detailed annotations. Other notable updating databases include the Biological Magnetic Resonance Data Bank (31); the eggNOG resource for comparative genomics (32) which more than doubles the number of species covered; the InterPro protein family compilation (33) which benefits from an improved interface that includes features inspired by the now-retiring Pfam website (34); and UniProt (35) which also has a redesigned website. The UniProt paper features interesting updates on the parallel and complementary annotation activities centred respectively on community curation and automatic rules-based methods.

In the section for metabolism, signalling and enzymes, REBASE, the popular database of restriction and modification enzymes returns (36) with genome coverage expanded tenfold and systems based on methylation having seen particularly dramatic growth. Other returning enzyme-focused databases include dbCAN_seq (37) which expands to include microbiome-derived carbohydrate-active enzymes, their encoding in gene clusters, and the prediction of substrates. The SulfAtlas database appears in NAR for the first time (38) and - recognising the increasing pace of research in the area and having demonstrated the reliability of the approach - switches to HMM-based (sub-)family assignment from fully manual updating. Meanwhile, the new database MeDBA (39) offers a welcome compendium of information on metalloenzyme sequences, families, structures and interactions. Finally on enzymes, GotEnzymes (40) predicts turnover numbers for 25 million enzyme-substrate pairs using AI methods. Elsewhere, the cornerstone resource in pathway analysis KEGG (41) contributes an update reporting on new genome and taxonomy browsers, while the equally foundational database STRING (42) reports on new co-expression data sources, improved interaction confidence estimates and the ability to process whole new genomes. Other well-used returning databases include MIBiG, whose update paper (43) has an interesting focus on annotation, including online ‘annotathons’; and SIGNOR (44) which has new data, a new interface, and new links to related projects focusing on diseases, including COVID-19. Finally, the new CovInter database (45) captures data on interactions between Coronavirus RNAs and host proteins.

The section on microbial and viral genomics leads off with a paper from the newly formed Bacterial and Viral Bioinformatics Resource Center (46), the successor to no fewer than three individual databases familiar to NAR readers - PATRIC (47), IRD (48) and ViPR (49). Resources hitherto confined to either bacterial or viral databases have been made available to both communities. In a similar space, the IMG/M and IMG/VR databases, for microbes and viruses respectively, each contribute updates (50,51). New features of the former include an improved genome context viewer while the latter has fresh tools for detection of metagenome-derived viral genomes and prediction of their hosts. The update from the popular MGnify resource for metagenomics data (52), recognising the scale and continued growth of the database, reports interestingly on the adoption of Deep Learning methods to annotate protein sequences with Pfam families (53). The importance of the microbiome to the colonised host is demonstrated by two databases. The returning GutMDisorder database (54), focused on human and mouse gut microbiomes, associates microbes with phenotypes and therapeutic interventions, now including data-derived links as well as those curated from the literature; while the new arrival CRAMdb (55) compiles microbiome data for an impressive 500 different animal species and offers sophisticated facilities to compare between different body locations or between different animals. Elsewhere, fungi are covered by a new database of fungal core genes UFCG (56) enabling easy and automated phylogenetic analysis of the kingdom; and the Fungal Names resource for fungal taxonomy (57) which also includes information on specimens, culture collections, publications and so on. Finally, an intriguing new arrival is ProPan (58), a resource for prokaryotic pan-genomes that allows for inference of core and dispensable genes across isolates of 1500 prokaryotes with implications for understanding of environmental adaptation and genome dynamics.

In the human, model organism and comparative genomics section, the Issue's second Breakthrough Article describes CEDAR, the Cancer Epitope Database and Analysis Resource (59). A companion database to the hugely popular Immune Epitope Database (IEDB; (60)), and borrowing its carefully standardised protocols, CEDAR covers cancer epitope and receptor data curated from the literature. Notably, the CEDAR authors consulted cancer immunology experts before designing the new user interface and the new database will significantly support resurgent interest in antigen-based immunotherapies. Also in the immunology area, the new PGG.MHC database (61) majors on the population genetics of HLA, especially in Asia; while updates from the heavily used IPD-IMGT/HLA include a refreshed website (62). Other major resources updating include Ensembl (63), the UCSC Genome Browser (64) and GWAS Central (65). Ensembl is recognising the increasing availability of multiple high-quality sequences for model species and now offers its first human pangenome graphs, but has also doubled the number of genomes covered, reaching out across species lacking transcriptomics data by employing alternative tools. New tracks at the UCSC Genome Browser are focused particularly on clinical annotations and single cell RNA-seq data, while the SARS-CoV-2 browser continues to be regularly updated with new variants of concern. The new COMBATdb database (66) covers the blood multi-omics of COVID-19 patients compared to healthy controls and will aid in the identification of biomarkers and therapeutic targets. A number of new databases focus on single cell transcriptomics particularly for human. HTCA (67) and HUSCH (68) each offer data on millions of single cells and rich suites of analytic tools while ABC Portal (69) focuses on blood cells, with relevance to blood cancers, and AgeAnno (70) majors on aging and additionally integrates information on chromatin accessibility and transcription factor binding. The ambitious SPEED (71), in contrast, looks across 127 species and multiple modalities of single cell data, allowing for sophisticated comparative analyses. Cell identity and cell fate during development are covered by several resources. The popular returning CellMarker (72), for human and mouse, reports significantly expanded content, both in terms of number and type of marker but also encompassing new sequencing technologies; while the new Cell Taxonomy database (73) covers thousands of curated cell types across 34 species. Another new database TEDD (74) looks at temporal (co-) expression and chromatin accessibility during development in model organisms while Lineage Landscape (75) explores similar themes, additionally covering epigenomics data. Finally, interesting perspectives on genome evolution are offered by the returning HGTree (76), now covering horizontal gene transfer in eight times as many prokaryotic species, and the new DupScan (77) which provides detailed insights into whole genome duplications in vertebrates.

As usual, cancer has a strong presence in the section on human genomic variation, diseases and drugs. The returning canSAR database (78), which reports updates including druggability assessments using AF2 models, is joined by the ASCancer Atlas (79) that focuses on oncogenic splicing events, including annotation of upstream regulators and downstream impact; and by CREAMMIST (80), which offers an improved integrative understanding of cancer cell drug responses. Two new databases, BIC (81) and microbioTA (82) recognise the importance of the microbiota in cancer, profiling composition and abundance at different sites in comparison with controls. For drug development PubChem is a foundational resource and its update here (83) reports on 120 new data sources, with patent coverage particularly strengthened. In the same area ChemFOnt (84) brings a new hierarchical ontology describing functions of biologically important chemicals. Major returning resources for drugs and their targets include DrugCentral (85), which now covers veterinary drugs too and has new data on adverse drug events, and TCRD/Pharos (86) which has incorporated fresh data sources and new data visualisations such as clever circular treemaps to intuitively map expression onto ontologies. The toxicological properties of drugs and other chemicals are covered by the popular CTD (87) which introduces ‘CTD tetramers’, information blocks containing chemical, gene, phenotype and disease compiled from pairwise interactions; and the newcomer TOXRIC (88), which is especially notable for offering standardised benchmarks ready for use by Machine Learning methods. An interesting newcomer in the pharmacology area is DRESIS (89) bringing superbly comprehensive coverage of drug resistance mechanisms. Elsewhere, inferring the potentially pathogenic consequences of sequence variants remains a major preoccupation. In this space FAVOR (90) is a significant new arrival, which comes accompanied by the FAVORannotator software, whose efficiency makes it an appealing option for cloud settings; while CIViC (91), publishing in NAR for the first time, focuses specifically on cancer and reports success in engaging with relevant communities for crucial ongoing curation alongside an appeal for more editors to step forward.

The final sections cover plants and then databases not comfortably accommodated elsewhere. CuGenDB (92) covers cucurbits, such the important crops cucumber and melon, and contributes an update paper reporting a new focus on detection and annotation of genomic variants, as well as improved gene expression options. Also on crops, the new CottonMD (93) compiles multi-omics information including metabolomes facilitating the identification of trait-linked features and speeding future plan breeding efforts. Nod-like receptors (NLRs) are key proteins in plant disease resistance and the new NLRscape (94) offers resources and analyses of sequences, families and structures (from AF2). A notable new arrival in NAR included in the last section is EMPIAR (95). Central to storing the raw data behind cryo-EM experiments, EMPIAR also archives volume EM and X-ray tomography images and provides visualisation tools for them. Further popular databases reporting updates this year include the Chemical Probes Portal (96) which has quintupled in size since first publication, and ProteomeXchange (97), the consortium of high-throughput proteomics resources, which celebrates 10 years. Finally, LitCovid reports an update (98) on its efforts to capture and curate the literature on COVID-19 with new emphasis on long COVID-19, variants and vaccines; while the collection of 3000 culture media for 40 000 microbial strains at Media*Dive* (99) will undoubtedly prove invaluable for microbiologists.

## NAR ONLINE MOLECULAR BIOLOGY DATABASE COLLECTION

For this 30th release of the NAR online Molecular Database Collection (as usual freely available at http://www.oxfordjournals.org/nar/database/c/), we have detected a problem with a number of entries which have been updated accordingly as part of our ongoing curation process, with regards to the latest version, we have updated 463 entries, 92 new resources have made it to the database and further 96 discontinued were removed, bringing the total collection to 1764 databases. We do appreciate the feedback as some errors can be present in older entries: thanks to the community we have identified a number of spurious entries which were updated. Our ongoing effort to ensure an up-to-date resource relies as well on the continuous scanning or current entries to detect discontinued services as well as scanning for new entries. We encourage authors to submit their updates to XMF at xose.m.fernandez@gmail.com in plain text, ideally according to the template found in http://www.oxfordjournals.org/nar/database/summary/1.
